# Evaluation of the eating habits of breast cancer patients

**DOI:** 10.12669/pjms.36.7.2368

**Published:** 2020

**Authors:** Tuba Kayan Tapan, Zeynep Erdogan Iyigun, Serkan Ilgun, Vahit Ozmen

**Affiliations:** 1 Dr. Tuba Kayan Tapan Nutrition and Dietetic, Faculty of Health Science, Demiroglu Bilim University, Istanbul, Turkey; 2 Dr. Zeynep Erdogan Iyigun Physical therapy and rehabilitation, Faculty of Health Science, Demiroglu Bilim University, Istanbul, Turkey; 3 Dr. Serkan Ilgun General Surgery Faculty of Medicine, Demiroglu Bilim University, Istanbul, Turkey; 4 Dr. Vahit Ozmen General Surgery Faculty of Medicine Istanbul University, Istanbul, Turkey

**Keywords:** Breast Cancer, Nutrition, Recurrence, Diet compliance

## Abstract

**Objective:**

To determine the relationship between the dietary characteristics of breast cancer patients.

**Methods:**

Patients with breast cancer whose treatments have finished and are in remission formed the study group and healthy people formed the control group. Demographic, anthropometric characteristics, food consumption frequency form and exercise status were recorded with all groups. Data analysis was done by SPSS 22.

**Results:**

In the study group, mean carbohydrate percentage was lower, while fat, fat percentage, monosaccharide, glucose, fructose, omega3(n3), saturated fatty acids(SFA), monounsaturated fatty acids (MUFA), vitamin A, C, E, B6, biotin and copper values were significantly higher (p<0.05). Recurrence was observed in seven patients (7.1%) during the follow-up period, hormone receptor levels (ER) and vitamin B2 intake (accuracy 93.9%) were inversely related to the recurrence of the disease (p=0.02).

**Conclusions:**

While the percentage of carbohydrate taken was lower in study group; total fat, n3, SFA, MUFA, monosaccharide, glucose, fructose, water-soluble fiber, B6, biotin and copper values were higher. Further studies are needed for vitamin B2 deficiency in patients with recurrence

## INTRODUCTION

Breast cancer is the most common cancer and the cause of death in women.^1^ In recent years, there has been a significant increase in disease-free and overall survival with early diagnosis and modern treatments.^2^ This increase naturally requires protection from other diseases and their complications for a healthier life.^3^ The relationship between obesity and sarcopenia and mortality was found in patients receiving breast cancer treatment. Therefore, nutrition during the treatment and follow-up period gains importance both in terms of preventing sarcopenia and weight control. Patients are required to meet 45-65% of their daily energy from carbohydrate, 20-35% from fat and 10-35% from proteins during and after chemotherapy. These rates can be achieved by consuming five portions of vegetables and fruits, 120-150 grams of meat and two cups (200 ml) of milk and milk products per day. In a study of Brown et al., it has been shown that regular consumption of vegetables and fruits and exercise programs together increase survival rates. However, there are also studies showing that consumption of vegetables and fruits has no effect on survival.4,5 Although there is different information about the effect of diet on survival in patients diagnosed with breast cancer, it is known that healthy and balanced diet has a positive effect on the quality of life and general well-being of individuals.

It has been observed that the diet content of breast cancer patients decreases and changes during their treatment. This is due to factors such as the prevalence of the disease, associated digestive system disorders, increasing nutritional requirements and incorrect diet practices by patients or health professionals.^6^ This study aimed to investigate the relationship between dietary characteristics and tumor characteristics of breast cancer patients and investigate the compliance of patients with dietary recommendations.

## METHODS

In November 2019-January 2020, at the Istanbul Florence Nightingale Hospital Breast Health Center, and people without randomly selected. The study group consisted of 98 patients who completed breast cancer treatment and followed in remission and the control group, was 89 volunteer women with similar demographic characteristics with no cancer history. Cross sectional study was designed. Both were 18 years old and above. There are limitations of being diagnosed with breast cancer in the study group, and not having breast cancer diagnosis in the control group, and not using psychiatric drugs. All participants were interviewed face-to-face, recording the demographic characteristics (gender, age, marital-educational status), body mass index (BMI). The frequency of nutrient consumption form^7^ was filled by themselves in to determine the average daily energy and nutrient consumption amounts in diet and physical activity status was evaluated (≥100 min/w were considered to exercise regularly). Before the study, patients were informed about portion control training. Height and weight measurements were carried out with Seca® (Medizinische Messsysteme und Waagen/Hamburg Deutschland.) electronic weighing system. BMI was classified with World Health Organization classification (≤19.9; =20- 29.9; and ≥30).^8^ The data obtained from the nutrient consumption frequency form was evaluated with the full version of Nutrition Information Systems 7.2. The calculated nutrient values were calculated with the Dietary Reference Intake (DRI) system adapted to gender and age.^9^ Patients who meet 67% of the references were classified inadequate, 67%-133% were sufficient and ≥133% were overconsumption.^10^ Istanbul Bilgi University Ethics Committee; 2019-40016-148 with Number and the date is five November 2019.

### Statistical Methods

SPSS 22 (IBM) software was used for statistical analysis. Distribution analysis of data was Kolmogorov-Smirnov, non-parametric independent data was the Mann-Whitney U, parametric data was Student T-test, dependent data was Wilcoxon test performed with. In the analyses, p<0.05 was accepted as significant.

## RESULTS

When the groups were compared in terms of age and BMI, comorbid diseases (diabetes, hyperlipidemia, coronary heart disease and hypertension) and smoking, no significant difference was found between the groups. The study group was found to do more exercise (Table-I).

**Table-I:** Demographic characteristics of the study and control group.

Demographic Characteristics	Study Group (n=99)	Control Group (n=89)	p
Age (median(min-max))	47.5 (24-79)	46 (23-79)	0.074^a^
BMI(median(min-max))	26.5 (17.7-43)	27.1(17.5-31.1)	0.87^a^
Internal disease (n(%))	32 (32.3)	35 (39.3)	0.34^b^
Exercise habit (n(%))	59 (59.5)	24 (26.9)	0.00^b^
Smoking (n(%))	6 (6)	9 (10.1)	0.49^b^

a student T-test,

b chi-square test

When the patients were divided into four groups according to their BMI (≤19.9; =20-24,99, =25-29,99 and ≥30), the groups were similar (P=0.693). Three of the patients received neoadjuvant and 96 of them received adjuvant chemotherapy. The mean weights at the end of the follow-up period of 45 months (±31, 7 months) and the weights measured at the time of diagnosis were found to be the same (69±13 kg vs 68.3±12.59 kg, p=0.352). The amounts are different, but p ratio is not different. (Table-I).

In the comparison of the average consumption amounts of both groups with the daily requirements; carbohydrates were taken less than the amount required in all. However, the daily amount of energy required to be taken from fats as well as vitamins A, E, B_2_, B_12_, E and C, magnesium, zinc, and niacin were taken more than required. In the study group, the percentage of energy coming from fat and vitamins A, E, B_2_, B_6_, C, B_12_ were taken more than the daily required amount according to the DRI recommendations. In the control group, the percentage of energy coming from fat and vitamins A, B_1_, B_2_, B_6_, C, B_12_ were taken in excess according to DRI recommendations. However, when the groups were compared, the daily intake of total fat, n_3_, SFA and MUFA were higher in the study than the control group (Table-II).

**Table-II:** Comparison of daily nutrient consumption between study and control groups.

Energy and Nutrients	Study Group χ̄	Control Group χ̄	p	Energy and Nutrients	Study Group χ̄	Control Group χ̄	p
Energy(kcal)	2221.066	2066.219	0.226	Vitamin B_1_(mg)	1.26	3.837	0.311
Carbohydrate(g)	193.631	209.019	0.14	Vitamin B_2_(mg)	1.802	1.78	0.895
Carbohydrate(%)	35.96	41.247	0	Vitamin B_6_(mg)	1.928	1.76	0.034
Protein(g)	90.575	88.404	0.689	Vitamin B_12_(mg)	7.93	6.526	0.554
Protein(%)	16.848	17.382	0.07	Niacin(mg)	29.045	30.624	0.265
Fat(g)	117.982	94.887	0.002	Biotin(mg)	65.011	56.199	0.01
Fat(%)	46.758	41.213	0	Vitamin C(g)	199.213	159.866	0.042
Cholesterol(mg)	347.66	346.031	0.925	Vitamin D(mg)	1.612	1.56	0.919
SFA(g)	36.139	31.004	0.056	Vitamin E(mg)	22.66	18.142	0.049
MUFA(gr)	49.922	36.517	0	Cupper(mg)	2.536	2.115	0.002
PUFA(g)	24.583	20.213	0.135	Calcium(mg)	954.745	1050.774	0.083
Omega_3_(g)	2.813	2.294	0.007	Magnesium(mg)	450.789	429.299	0.471
Omega_6_(g)	21.767	18.128	0.12	Potassium(mg)	3561.999	3355.741	0.171
Monosaccharides(g)	40.849	26.766	0	Selenium(mg)	0.082	0	0.338
Galactose(g)	1.859	3.036	0.683	Sodium(mg)	2615.534	2448.144	0.945
Fructose(g)	19.929	14.568	0.002	Zinc(mg)	12.961	13.02	0.867
Glucose(g)	17.958	14.107	0.005	Fiber(g)	33.304	32.236	0.28
Vitamin A(mg)	1858.936	1494.68	0.05	Water soluble fiber(g)	11.215	10.136	0.049
Carotene(mg)	3.055	3.354	0.492	Water(ml)	1461.787	1387.821	0.362

*p<0.05

Total 58 patients (58.6%) stated that they changed their dietary habits after the diagnosis of breast cancer. However, no significant difference was found in consumed amounts between the groups that changed and did not change the dietary habits (p>0.05).

When the use of dietary supplements of the groups were examined, 45 (45.9%) individuals in the study and 37 (41.5%) in the control group were using dietary supplements. Vitamin D, turmeric, ginger, and propolis were used. There was no difference between the groups in terms of usage (p=0.55). No significant difference was between BMI and tumor size, ER, Ki_67_, molecular subtypes of the tumor and nutrient consumption rates.

In the study group (median 45 months (14-96) follow-up), local or systemic recurrences were observed in seven individuals. No significant difference was found in the treatments, disease stages, progesterone receptor, Ki_67_, ER ratios, BMI, age and exercise habits between patients with and without recurrence (p<0.05). Between with and without recurrence, a significant difference was found in the amount and percentage of daily protein, water amount, vitamin A, galactose, n_3_, biotin, B_1_, B_2_ and niacin intake. In the logistic regression model, where these different parameters and the ER ratio which is also different between the two groups were included, vitamin B_2_ was determined as independent variables (p=0.02). In the multivariate reduced model (accuracy 93.9%), the ER rate and the vitamin B_2_ taken in the diet were inversely correlated with the systemic or local recurrence of the disease (OR=0,19 is low effective, OR=0,97 is high effective) (Table-III).

**Table-III:** The effect of diet on disease recurrence.

	Univariate Model				Multivariate Reduced Model			

	OR	95% confidence interval		p	OR	95% confidence interval		p
ER rate	0.97	0.95	0.99	0.02	0.97	0.96	0.99	0.02
Water(ml)	0.99	0.99	1	0.03				
Protein(g)	0.97	0.94	0.99	0.02				
Protein(%)	0.79	0.57	0.97	0.031				
Biotin(mg)	0.96	0.92	0.99	0.038				
Vitamin A(mg)	0.99	0.99	1	0.031				
Vitamin B_1_(mg)	0.12	0.01	0.81	0.03				
Vitamin B_2_(mg)	0.17	0.04	0.66	0.01	0.19	0.46	0.82	0.02
Galactose(g)	0.38	0.15	0.97	0.04				
Omega _3_(g)	0.36	0.14	0.9	0.02				
Niacin(mg)	0.9	0.86	0.99	0.04				

OR: odds ratio, ER: estrogen receptor ratio

## DISCUSSION

Studies have shown that high BMI is among the risk factors for breast cancer.^11^ Therefore, weight control is recommended to patients after diagnosis and patients with this recommendation vary. In the literature, compliance with the weight control recommendation of patients after breast cancer is reported as 58-77%.^12^ In this study, when the mean BMI of patients was taken, no significant difference was found between pre and post-diagnostic.

Poor dietary habit characterized with highly refined sugar and both saturated and trans fat consumption, low n_3_, natural antioxidants, and fiber intake, appears to be linked to increased risk of breast cancer and death by modulating inflammation. On average, obesity increases the mortality risk by approximately 30%. In another study, it was reported that obesity increased the risk of breast cancer by 35-40%.^13^ The fact that women often gain weight after the diagnosis of breast cancer also increases the risk of death and survival. Not only dietary habits but also body weight control should be given more importance.^14^

There is a desire to change lifestyles and dietary habits in patients diagnosed with cancer. In this study, 58.6% of the patients changed their diet after diagnosis with the recommendation of a doctor or dietician. In Malaysian, 57.8% of the patients reported that they changed their diet at a similar rate to our study.^15^ In a meta-analysis, dietary change after diagnosis was to reduce energy intake, increase vegetable and fruit consumption, to increase fiber intake and improve dietary quality. However, in our study, these changes didn’t make any difference in weight and BMI. After the diagnosis of breast cancer, the use of food supplements is also increasing. In a study, the use of nutritional supplements was 56% before, 62% after diagnosis. The most commonly used supplements were found to be fish oil, multivitamins and minerals and evening primrose oil.^16^ In our study, taken food supplements did not increase significantly after the diagnosis compared to the control group (p>0.05). Taken food supplements are identified as n_3_, vitamin D, green tea and ginger.

In this study, the ratio of energy from carbohydrates was found to be 36%. Although a low carbohydrate diet or a ketogenic diet is recommended in breast cancer patients, evidence supporting the effect of these diets on survival is insufficient.^17^ It was determined that breast cancer patients had a higher rate of fat intake and fat percentage (46.7%) was higher than carbohydrates (35,9%). However, studies showing that although weight loss is observed in individuals fed with a low-fat diet, it does not affect survival in breast cancer.^18^ In a study, 48,835 postmenopausal women, it was proved that a low-fat diet reduces the risk of dying from breast cancer for a postmenopausal woman.^19^

Among women, n_3_, PUFA intake was found to be inversely related to breast cancer risk and mortality.^20^ In our study, the average daily intake of n_3_ (2.8g/day) increased due to the excess amount of fish, green leafy vegetables, and oilseeds consumed weekly. The amount of MUFA(49.92%) was high, due to the high amount of olive oil taken on a daily diet. The total fat intake is high, because the high consumption of natural butter and fish. Breast cancer risk is positively correlated with SFA, n_3_/n_6_. N_3_-rich diet, increased fish and reduced n_6_ consumption reduce the risk of breast cancer.^21^ It is stated that n_3_/n_6_ ratio of diets rich in vegetables and fruits (Mediterranean diet), physical activity, and low BMI are effective in preventing breast cancer.^22^

High plasma vitamin B_6_ and B_2_ can reduce the risk of breast cancer especially in premenopausal women and also has a protective effect against breast cancer.^23^ ER rate and the amount of vitamin B_2_ taken in the diet were found to be inversely correlated with the systemic or local recurrence of the disease, in this study (p=0.02).

### Limitations of the study

The number of studies about the dietary habits of breast cancer patients in Turkey is limited. There is no similar study. The compliance of the treated breast cancer patients to oncologist and dietician recommendations resulted in less consumption of carbohydrate (bread), but more frequent consumption of whole wheat flour, rye, oat bread, leading to higher vitamin B_6_ intake. High frequency of fish, nuts, and olive oil, natural butter consumption increase the amount and percentage of fat. In our study, when study group, with and without recurrence are compared; the ER rate and the vitamin B_2_ were found to be different between two groups (study group>control group) and identified as independent variables.

## CONCLUSION

BMI values remained the same in patients receiving breast cancer treatment, although they adhered to a healthy diet and regular exercise recommendations. According to our study, increasing the ratio of vitamin B_2_ in the diet may contribute to the prevention of recurrences, but more patients and more comprehensive prospective clinical studies are needed in this front.

### Authors’ Contribution:

**TKT:** Conceived, designed, data collection, editing of the manuscript and takes the responsibility and is accountable for all aspects of the work in ensuring that questions related to the accuracy or integrity of any part of the work are appropriately investigated and resolved. **ZEI, SI:** Conceived, designed and did statistical analysis & editing of manuscript. **VO:** Critical Review and final approval of manuscript.
